# A randomized, double-blind water taste test to evaluate the equivalence of taste between tap water and filtered water in the Taipei metropolis

**DOI:** 10.1038/s41598-020-70272-y

**Published:** 2020-08-07

**Authors:** Jing-Rong Jhuang, Wen-Chung Lee, Chang-Chuan Chan

**Affiliations:** 1 Institute of Epidemiology and Preventive Medicine, College of Public Health, National Taiwan University, Rm. 536, No. 17, Xuzhou Rd., Taipei, 100 Taiwan; 2 Innovation and Policy Center for Population Health and Sustainable Environment, College of Public Health, National Taiwan University, Taipei, Taiwan; 3 Institute of Occupational Medicine and Industrial Hygiene, College of Public Health, National Taiwan University, Rm. 722, No. 17, Xuzhou Rd., Taipei, 100 Taiwan

**Keywords:** Environmental social sciences, Energy and society, Psychology and behaviour, Sustainability

## Abstract

High water quality and sufficient water availability are the main concerns of water users. Promoting the efficient use of tap water can contribute to sustainable drinking water management and progress towards Sustainable Development Goals. In many metropolises, water suppliers treat municipal water with appropriate treatment processes and well-maintained distribution infrastructure. Under this circumstance, it is acceptable that municipal water can be a source of drinking water. The presence of residual chlorine in tap water, connected to municipal water supply, inactivates pathogenic microorganisms and prevents recontamination. However, adding chlorine to tap water may affect the organoleptic properties of drinking water. On the other hand, the use of point-of-use (POU) water dispensers, which provides an additional treatment step on tap water, is not energy-efficient. A randomized, double-blind water taste test was conducted in the Taipei metropolis to assess whether tap water from public drinking fountains and filtered water from POU water dispensers have similar organoleptic properties. An odds ratio (OR) and the area under the receiver operating characteristic curve (AUC) were used to measure the participants’ ability to distinguish between the two water varieties. A five-region hypothesis test was conducted to test the OR, and a 95% bootstrap confidence interval of the AUC was calculated. The results of the study showed that the 95% five-region confidence interval of OR equal to (0.5, 1.49), and the 95% bootstrap confidence interval of AUC equal to (0.42, 0.56). These results implied that people in the Taipei metropolis could not distinguish between tap water and filtered water. It is recommended that more drinking fountains be installed and maintained fully functional and clean to achieve excellence in tap water access.

## Introduction

High water quality and sufficient water availability are the main concerns of water users. Water utilities must treat and supply water to meet specific water quality standards. In many metropolises, water suppliers treat municipal water with appropriate treatment processes and well-maintained distribution infrastructure, ensuring high-quality municipal water and sufficient water availability. Under this circumstance, it is acceptable that municipal water can be a source of drinking water. Tap water, connected to municipal water supply, is a common and efficient source of drinking water. The presence of residual chlorine in tap water inactivates pathogenic microorganisms that cause waterborne diseases^1,2^ and prevents recontamination during storage or transportation^3^. The World Health Organization (WHO) provided guidelines for drinking-water quality that residual chlorine levels in tap water should be maintained at concentrations of 0.2–5 mg/L^4^.

The United Nations General Assembly has proposed Sustainable Development Goals (SDGs) to achieve a more sustainable future by 2030^5^. Among the 17 goals, SDG 6 addresses the availability and sustainable management of water and sanitation. Promoting the efficient use of tap water can contribute to sustainable drinking water management and progress towards SDG 6. However, adding chlorine to tap water exhibits effects on the taste and odor of drinking water, which can reduce people’s preference for tap water^6–8^ and impede acceptance and sustainability of the water quality intervention^9^. The point-of-use (POU) water dispenser, which works by connecting to municipal water supply and drawing water from the waterline that is already in place, provides an additional treatment step on tap water. The application of replaceable filter in a POU water dispenser can improve the organoleptic quality of tap water^10–12^ by removing chlorine, solid precipitates, discoloration, unpleasant scent. A POU water dispenser has the option to provide hot or cold water on command. And the predominant demand for energy in such water dispensing systems is from the heating or cooling of water before consumption. In Taiwan, the total energy consumed by 5.48 million water dispensers was 3.15 billion kWh per year^13^. The water dispenser was also the fifth electricity-consuming household appliances in Taiwan^14^. High energy consumption can complicate the achievement of SDG 7, which represents affordable and sustainable energy. Also, in a city, tap water and water from POU water dispensers are connected to the municipal water supply, from the same water source, water treatment processes, and distribution piping system. For sustainability in water supply, it is unnecessary to treat water that is already of good quality at the end-user point. Therefore, a better understanding of the public perception and preferences of tap water can contribute to improvements in water management, consumer services, and sustainability.

Municipal water in Taipei city meets drinking-water standards in WHO, USA, Europe, and Japan^15^. The perception and preferences of tap water are still unknown in the Taipei metropolis. The study aimed to investigate whether tap water has organoleptic properties similar to filtered water from POU water dispensers. It was expected that people could not distinguish the two water varieties such that there is no advantage in treating water that is already of good quality at the end-user point.

## Material and methods

### Study design and randomization

**Figure 1:**
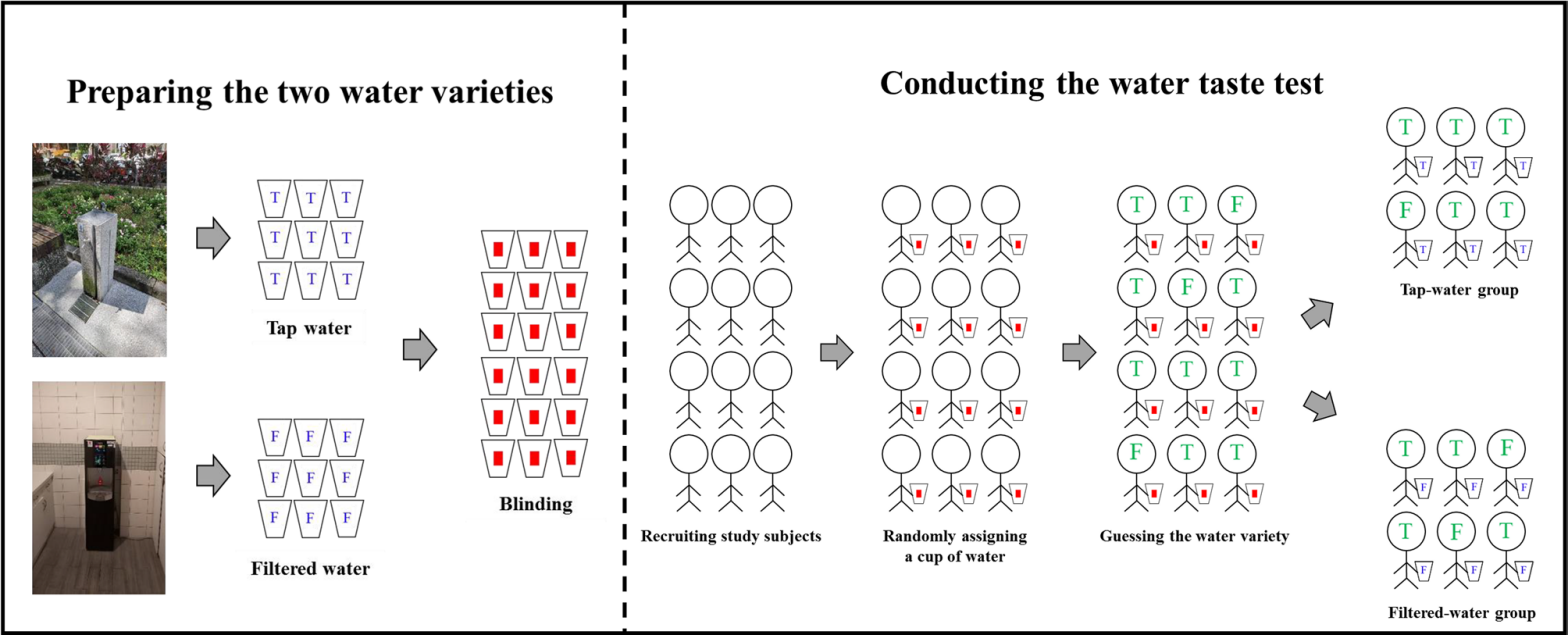
Procedure for the water taste test.

A randomized, double-blind water taste test was designed (Fig. 1). Water from a public drinking fountain (tap water) and cold water from a POU water dispenser (filtered water) were obtained and were let stand for an hour at room temperature. A thermometer was used to ensure that the temperature was at 25 °C for both the water varieties. Paper cups with the same appearance were prepared. For each paper cup, a random decimal number between 0 and 1 was generated by using a computer, and the cup was assigned to the tap-water group if the number was ≥ 0.5 and to the filtered-water group if the number was < 0.5. A fixed and identical amount (200 ml) of the appropriate water variety was poured into each paper cup according to the group to which it was assigned. A sealed letter containing the group information was attached to the outside of each paper cup.

One-on-one interviews were conducted. The interviewer, who was not involved in water sample preparation, first told the participant that residual chlorine exists in tap water from public drinking fountains but not in filtered water from POU water dispensers. Next, the participant was invited to taste a cup of water. (Participants who refuse to drink the water were excluded.) Neither the interviewer nor the participant knew which water variety was served in the cup, except that it could be tap water or filtered water with equal probability. The interviewer then instructed the participant to guess the water variety. After the guess, the participant opened the sealed letter to reveal the correct answer.

### Measures of distinguishability

The participants’ ability to distinguish between the two water varieties was measured using an odds ratio (OR)^16^ as follows:

1 $$\hbox{OR}=\frac{\hbox{Se}}{1-\hbox{Se}}\times \frac{\hbox{Sp}}{1-\hbox{Sp}} ,$$

where Se (sensitivity) and Sp (specificity) represent the probabilities of guessing the correct answer in the tap-water group and the filtered-water group, respectively. When obtaining OR = 1, it indicated that the proportions of the participants guessing “tap water” were equal in the two groups; that is, the participants could not distinguish between the two water varieties by any means. When obtaining OR > 1, it indicated that the proportion of the participants replying “tap water” was higher in the tap-water group than in the filtered-water group. The higher the OR, the stronger the participants’ abilities to distinguish between the water varieties. When obtaining OR < 1, it indicated that the participants were not only unable to distinguish between the water varieties but also tended to guess incorrectly. The smaller the OR, the stronger the tendency to guess incorrectly.

The participants' ability to distinguish between the water samples was also measured using the area under the receiver operating characteristic curve (AUC)^17^:

2 $$\hbox{AUC}=\frac{1}{2}\times \left(\hbox{Se}+\hbox{Sp}\right).$$

An AUC of 0.5 indicated that the participants could not distinguish the two water varieties by any means. An AUC of > 0.5 indicated that the participants could distinguish between the water varieties, and the higher the value, the stronger was their ability to distinguish between the water varieties. An AUC of < 0.5 indicated that the participants could not distinguish between the water varieties, and the smaller the value, the stronger was the tendency to guess incorrectly.

### Statistical analyses

A newly proposed five-region hypothesis test^18^ was conducted to test the OR. The five regions were defined as $\hbox{OR}>2$ (a recognizable ability to distinguish between the water varieties; we also consider a more lenient criterion of $\hbox{OR}>1.5$ for this category), $1< \hbox{OR} \le 2$ (a negligible ability to distinguish between the water varieties; also a stricter criterion of $1<\hbox{OR} \le 1.5$ for this category), $\hbox{OR}=1$ (no ability to distinguish between the water varieties), $0.5\le \hbox{OR} <1$ (a weak tendency to guess incorrectly), and $\hbox{OR}<0.5$ (a strong tendency to guess incorrectly). The 95% five-region confidence interval^18^ of the OR was also calculated. To conclude^18^ that the participants have no recognizable ability to distinguish between the two water varieties ($\hbox{OR}\le 2$), at least 207 participants were required to be recruited to achieve a power of 80% at a significance level of 0.05. Furthermore, we generated 10,000 bootstrap samples and calculated a 95% bootstrap confidence interval^19^ of the AUC. All analyses were performed with R version 3.5.2^20^.

### Consent for publication

Not applicable.

### Ethical approval and consent to participate

Not applicable.

## Study site, participants, and data collection

### Water supply and sanitation

There are two state-owned water utilities in Taiwan; Taiwan Water Corporation provides water supply to Taiwan except for the Taipei metropolis, whereas the Taipei Water Department is exclusively responsible for supplying water to the Taipei metropolis. The primary source of raw water is Xindian Creek, representing 97% of the total raw water supply in the Taipei metropolis. Qingtan Dam and Zhitan Dam are in operation at the Xindian Creek. The two water intake units take in 1.08 (Qingtan Dam) and 2.70 (Zhitan Dam) million cubic meters of raw water daily, respectively, which are conveyed with gravity via tunnels to the Zhangxingm, Gongguan, or Zhitan Purification Plants for treatment. The treatment process comprises testing, applying chemical disinfectants, coagulation, mixing, sedimentation, and filtering. Wastes discharged from the water purification process, including settled flocculating waste and filter backwash waste, are sent to Zhitan or Gongguan purification plants to process.

Water pipeline network with a caliber ranging from 75 to 3,400 mm that add up to a total length over 3,000 km has been placed in the Taipei metropolis. A water supply monitoring and control system was developed in 1991 for better control of pressure changes and leakage in the distribution system, flexible adjustment of water supplies, and early detection and prevention of accidents. Due to the requirement for adjusting the delivery of treated water to meet demand adequately, 92 distribution basins have been set up. Also, 60 pumping stations have been set up at appropriate locations to enable water supply to reach the farthest ends of the distribution piping system, particularly those at high altitudes.

Some measures have been implemented in the Taipei metropolis to meet the goal of sustainable water resources. Automatic Water Quality Monitoring System was established in 1985 to monitor the water quality in the raw water intakes, the treatment process at its purification plants, and the distribution system. To enhance water availability for drinking, approximately 280 public drinking fountains that provide clean and safe tap water have been installed^21^. Also, a QR code that provides water users with updated information about the quality of tap water (turbidity, pH, and residual chlorine) was equipped on each public drinking fountain.

### Participants and data collection

The study protocol was approved by the College of Public Health, National Taiwan University (NTU), where the study was conducted (in the Zhongzheng District of the Taipei metropolis). All methods were carried out following the guidelines and regulations of NTU. Students and teaching faculty members of the College of Public Health, NTU, were recruited for the study. Informed consent was obtained from all the participants. The study period was from March to April 2018. The primary source of drinking water in this study site (NTU Public Health Building) is the POU water dispensers. Currently, a public drinking fountain has been set up, which can be another choice for the students and the teaching faculty members to drink. The participants were invited to attend the water taste test in a small room, and after the test, they can win a gift. Eight well-trained interviewers collected data from the participants. The collected variables include gender and position of each participant, whether or not he (or she) had drunk cold water from water dispensers in the previous month and had drunk water from drinking fountains before, the water variety he (or she) guessed, the actual water variety he (or she) drank, and his (or her) preference for tap water from drinking fountains.

## Results

A total of 278 participants took part in the test; 139 were randomly assigned to the tap-water group and the remaining to the filtered-water group. Table 1 presents the baseline characteristics of the participants. The two groups did not differ significantly in their characteristics. Table 2 presents the results of the water analysis of the two water varieties. The water qualities of the two water varieties were similar except for total residual chlorine and pH.

**Table 1:** Baseline characteristics of the participants.

	Tap-water group	Filtered-water group	*P*-value
	Number of subjects (%)	Number of subjects (%)	
**Gender**			0.12
Male	52 (37.4)	66 (47.5)	
Female	87 (62.6)	73 (52.5)	
**Position**			1.00
Students	124 (89.2)	123 (88.4)	
Faculties	15 (10.8)	16 (11.6)	
**Having drunk cold water from water dispensers in the previous month**			0.80
Yes	93 (66.9)	96 (69.1)	
No	46 (33.1)	43 (30.9)	
**Having drunk water from drinking fountains before**			0.34
Yes	16 (11.5)	10 (7.20)	
No	123 (88.5)	129 (92.8)

**Table 2:** Results of the water analysis of the two water varieties.

Sampling date	2018/02/26		2018/03/05		2018/03/12		2018/03/19	
Sample name	Tap water	Filtered water	Tap water	Filtered water	Tap water	Filtered water	Tap water	Filtered water
Total residual chlorine (mg/L)	0.35	< 0.011	0.27	< 0.011	0.36	< 0.011	0.39	< 0.011
pH	7.4	9.4	7.4	7.9	7.4	8.5	7.3	8.4
Turbidity (NTU)	0.25	0.20	0.10	0.10	0.10	0.15	0.05	0.20
Total dissolved solids (mg/L)	62	71	62	66	71	63	64	72

Test method: Total residual chlorine, Spectrophotometry (MDL = 0.011 mg/L); pH, Electrode method; Turbidity, Nephelometry; Total dissolved solids, DS meters.

Table 3 presents the results of the water taste test. A total of 216 participants (77.7%) replied, “tap water.” The numbers of the participants who replied, “tap water,” were 106 (76.3%) and 110 (79.1%) in the tap-water group and the filtered-water group, respectively. The Se, Sp, and OR estimates were 0.76, 0.21, and 0.85, respectively. Table 4 presents the results of the analysis of the participants’ abilities to distinguish between the two water varieties. The 95% five-region confidence interval of the OR for all the participants was (0.5, 1.49), excluding $\hbox{OR}>2$ (and also $\hbox{OR}>1.5$) and $\hbox{OR}<0.5$ entirely; the p-value for the $\hbox{OR}>2$ hypothesis (a recognizable ability to distinguish between the water varieties) was 0.01 (0.02 for the $\hbox{OR}>1.5$ hypothesis), and the p-value for the $\hbox{OR}<0.5$ hypothesis (a strong tendency to guess incorrectly) was 0.03. These results indicated that the participants could not distinguish between the two water varieties and that the indistinguishability of the water varieties was statistically significant. Besides, the estimate of the AUC was 0.49, with a 95% bootstrap confidence interval of (0.42, 0.56), which encompassed the null value of 0.5.

**Table 3:** Results of the water taste test.

Responses	Tap-water group	Filtered-water group	Totals (%)
	Number of subjects (%)	Number of subjects (%)	
Replying tap water	106 (76.3)	110 (79.1)	216 (77.7%)
Replying filtered water	33 (23.7)	29 (20.9)	62 (22.3%)

**Table 4:** Results of the analysis of the abilities to distinguish between the two water varieties.

	Number of subjects	Odds ratio		Area under the receiver operating characteristic curve	
		Estimate	95% five-region confidence interval	Estimate	95% Bootstrap confidence interval
Totals	278	0.85	[0.5, 1.49]	0.49	[0.42, 0.56]
**Gender**					
Male	118	0.83	[0.45, 1.73]	0.49	[0.38, 0.60]
Female	160	0.91	[0.43, 2.00]	0.48	[0.39, 0.57]
**Position**					
Students	247	0.74	[0.43, 1.40]	0.46	[0.38, 0.54]
Faculties	31	1.50	[0.45, 4.96]	0.55	[0.37, 0.72]
**Having drunk cold water from water dispensers in the previous month**					
Yes	189	1.02	[0.52, 1.99]	0.50	[0.42, 0.59]
No	89	0.52	[0.21, 1.55]	0.42	[0.30, 0.55]
**Having drunk water from drinking fountains before**					
Yes	26	0.71	[0.17, 2.95]	0.46	[0.26, 0.66]
No	252	0.90	[0.50, 1.65]	0.49	[0.41, 0.56]

A subgroup analysis (Table 4) was also performed. The value of $\hbox{OR}>2$ was rejected in male participants, female participants, students, and those who have not drunk cold water from water dispensers in the previous month. The values of $\hbox{OR}>2$ and $\hbox{OR}<0.5$ were rejected in those who have drunk cold water from water dispensers in the previous month and those who have not drunk water from drinking fountains before. However, because of the small sample size, the power was insufficient to reject $\hbox{OR}>2$ or $\hbox{OR}<0.5$ in the faculty members and those who have drunk water from drinking water fountains before. The 95% bootstrap confidence intervals of the AUC encompassed 0.5 in all subgroups.

## Discussion

A randomized, double-blind water taste test was performed, and the results showed that the participants could not distinguish between tap water and filtered water. The participants (after the water taste test) were asked whether they were willing to drink from drinking fountains if they could choose to drink from POU water dispensers. Most of the participants (252, 90.6%) provided affirmative responses. Based on these findings, in general, it is unnecessary to treat municipal water in the Taipei metropolis at the end-user point.

In the water taste test, the participants were being told from the outset that the water to be drunk had a 50:50 chance of being from a drinking fountain and a water dispenser. However, the participants had a biased belief that the water was more likely to be from a drinking fountain than a water dispenser (78:22), perhaps because they tend to associate the taste of water dispensers with cold or hot water rather than room temperature water as in this study. Nevertheless, the OR, the primary measure in the study, is impervious to such a bias. The study aimed to prove the equivalence of the two water varieties on taste. A conventional hypothesis test can only prove nonequivalence; we cannot conclude that the taste of the two water varieties is equivalent when the test result is nonsignificant. By contrast, the five-region hypothesis test we used in this study is a legitimate test to conclude that the OR significantly fell into a pre-specified equivalence region (from 0.5 to 2.0; or from 0.5 to 1.5) of the two water varieties, which indicated the taste of the water varieties is statistically equivalent^22^.

In a group interview, participants may discuss the water tastes; therefore, in this study, a one-on-one interview was adopted to avoid possible contamination biases. In this study, students or teaching faculty members who had been smoking or eating within one hour before the water taste test were not excluded. However, the randomization was conducted to control any possible bias this may induce. In most settings, we believed that people would drink water from an easily accessible source to quench their thirst but would not drink deliberately from two different sources at the same time merely to compare the tastes. Therefore, each participant tasted only one water variety, unlike other studies, which let each participant taste no less than two water varieties^23,24^. In general, information about the characteristics of water samples is not to be given to tasters in a sensory evaluation test. In this study, information about residual chlorine exists in tap water was told before the water taste test because most of the participants have not drunk water from drinking fountains before, and the preference for tap water in the study site was unknown before the study.

Information about the chemical quality of the two water varieties would be crucial to evaluate the study results. According to previous studies, the taste detection thresholds for residual chlorine has an extensive regional variation, from 0.17 to 0.71 mg/L^4,24,25^. The residual chlorine levels of tap water ranged from 0.27 to 0.39 mg/L during the study. However, the two water varieties were allowed to stand for an hour at room temperature before the water taste test (for ensuring proper control). This procedure may allow some residual chlorine in tap water to dissipate and may have rendered the two water varieties more challenging to distinguish. An extreme pH value on filtered water was observed on one particular day in the study period, which may also influence the study results. A previous study^26^ indicated that it is difficult to discriminate the two water varieties when the difference in total dissolved solids (TDS) among the two is lower than 150 mg/L. In this study, there was a minor difference (about 10 mg/L) in TDS among the two water varieties during the study period. Additionally, minerals are correlated with the taste of water^27^ but were not measured in this study.

Bottled water, which is also an alternative to tap water^6,7,27–29^, was not compared in this study because whether consumers could perceive the presence of residual chlorine in drinking water was mainly concerned. Water samples in the study were only collected from a POU water dispenser and a public drinking fountain. Further studies can be conducted to validate our findings in other locations in the Taipei metropolis (internal validation) or other cities having similar water sources, treatment processes, and distribution piping systems (external validation). The study results could not be generalized and extrapolated to other water varieties with medium or high TDS or to consumers who are more sensitive to the residual chlorine level, for example, French consumers^25^, bottled water drinkers^25^, or professional water sommeliers.

Although a POU water dispenser can provide clean and safe drinking water to meet SDG 6, high energy consumption constitutes obstacles in achieving SDG 7 (affordable and clean energy). This problem exhibits a trade-off between SDG 6 and SDG 7^30,31^. By contrast, drinking tap water improves energy efficiency. In locations where tap water has acceptable quality at the end-user point, it is recommended the use of tap water for drinking to achieve the synergistic development of SDG 6 and SDG 7 by providing clean water with affordable energy. To drink hot or cold water, using kettle heaters and refrigerators are more energy-efficient than using water dispensers; the average electricity consumption by kettle heaters (14.38 kWh per month per household^32^) is lower than that by water dispensers (26.00 kWh per month per household^14^), and refrigerators are already in use in many households in Taiwan.

## Conclusion and perspectives

The study results concluded that people in the Taipei metropolis could not distinguish between tap water and filtered water. It is recommended that more drinking fountains be installed and maintained fully functional and clean^33,34^ to achieve excellence in tap water access. POU water dispensers with functions of either heating or cooling water managed by the government can be uninstalled or replaced with drinking fountains. Public education toward more tap water use should be implemented. Furthermore, risk indices^35^ for assessing the water supply systems should be determined to prevent substantial water quality deterioration. For achieving sustainable water management, we suggest using reclaimed water^36–38^ to balance water supply and demand.

## Data Availability

Data collected from the water taste test and R code for statistical analysis are available at https://github.com/yoyo830303/water-analysis. The Taipei Water Department provided data about the water quality analyses of the two water varieties.
